# Validity and reliability of Fried frailty phenotype in Turkish population

**DOI:** 10.3906/sag-2105-165

**Published:** 2021-10-23

**Authors:** Hacer DOĞAN VARAN, Olgun DENİZ, Süheyla ÇÖTELİ, Rana TUNA DOĞRUL, Muhammet Cemal KIZILARSLANOĞLU, Berna GÖKER

**Affiliations:** Division of Geriatric Medicine, Department of Internal Medical Sciences, Faculty of Medicine, Gazi University, Ankara, Turkey

**Keywords:** Fried, frailty, validation, Turkish

## Abstract

**Background/aim:**

Frailty is an important, multidimensional geriatric syndrome defined as increased vulnerability to stressors. Fried frailty phenotype (FFP) is one of the most widely used models to define physical frailty. The aim of this study is to investigate the cross-cultural validity and reliability of Fried frailty phenotype (FFP) in older Turkish population.

**Materials and methods:**

A total of 450 patients, aged 59 years and over, were included. FFP translated into Turkish was used. Hand grip strength cut-off values that best predict low skeletal muscle mass index (SMI) for Turkish men and women were calculated. A modified version of FFP was created by rescoring FFP according to these cut-off values applicable to Turkish population. Correlation analysis between the frailty assessment by comprehensive geriatric evaluation of clinician experienced in geriatric medicine, and FFP and modified version of FFP were performed for validation. Thirty-five patients underwent frailty assessment with FFP twice for reliability assessment. Inter-rater and intra-rater agreements were investigated.

**Results:**

Clinician’s decision of frailty status demonstrated significant agreement with the results of FFP, as well as modified FFP. Interrater and intra-rater compliance were good. Best hand grip strength cut-off values for predicting low SMI in older Turkish population were determined as ≤13.6 kg (AUC: 0.841, p < 0.001) for women and ≤27.7 kg for men (AUC: 0.779; p < 0.001). Modified FFP had a good agreement with the FFP.

**Conclusion:**

FFP is a valid and reliable tool for Turkish population.

## 1. Introduction

Frailty, a multidimensional clinical state defined as increased vulnerability to stressors, is an important geriatric syndrome that is known to be related with worse clinical outcomes like disability and mortality [1]. Detecting frailty status of older adults is essential to struggle with the related adverse health outcomes.

Concept of frailty first appeared in clinical geriatric literature in 1950s–1960s and in 2001 Fried and colleagues suggested the Fried phenotype to define physical frailty [2]. Considering that physical, social and cognitive status can affect frailty, until today, a great many frailty models based on different perspectives containing cumulative deficit or psychosocial vulnerability were developed to define frailty status of patients [3]. Among all these frailty models or tools, today there is no gold standard tool to detect frailty status of the patients.

Comprehensive geriatric assessment is considered as the gold standard method for determining frailty status of the patients. However, the clinician’s assessment of frailty by performing comprehensive geriatric evaluation is time consuming in busy clinical practice.

Fried frailty phenotype (FFP) is one of the most widely used models to define frailty in busy clinical practice and in clinical studies. It is based on physical frailty and contains 5 basic criteria including self-reported exhaustion, loss of weight, low physical activity, slow walking speed, and low grip strength [4]. In these criteria, hand grip strength cut-off values are defined as the expected values for the gender and body mass index (BMI) of the patient. However, expected cut-off values for the same gender and BMI might vary among populations.

The hypothesis of this study is that using population specific hand grip strength cut-off values might be more appropriate for detecting frailty with FFP and population specific cut-offs might increase the accuracy of FFP. Therefore, the primary aim of this study is to determine the hand grip cut-off values for Turkish population which are the best to predict low skeletal muscle mass index (SMI) according to gender.

The secondary aim of the study is to evaluate the validity and reliability of the FFP and modified FFP (modified FFP was created by rescoring FFP by using the Turkish population specific hand grip strength cut-offs by gender) via using the gold standard, i.e. frailty status clinically defined by the expert geriatrician after performing comprehensive geriatric assessment.

## 2. Materials and methods

### 2.1. Patients and the procedure

Patients, aged 59 years and older admitted to a geriatric outpatient clinic, were invited to participate to the study, consecutively. Those who did not cooperate enough to answer the questions or could not follow the requested instructions and patients who were not eligible for bioelectrical impedance analysis (who have pacemaker, metal implant, peripheral edema) were excluded from the study. Finally, a total of 450 patients were included in this study. Informed consent was obtained from each patient prior to the study entry.

Age, gender, number of drugs, alcohol use, smoking status and comorbid diseases of the patients were recorded, and anthropometric measurements including height, weight, and calf circumference were performed. Each participant underwent comprehensive geriatric assessment involved application of the questionnaires of Katz activities of daily living (ADL)[5, 6], Lawton Brody instrumental activity of daily living (IADL) [7], standardized MMSE [8, 9], Yesavage geriatric depression scale short form (GDS) [10, 11], mini nutritional assessment short form (MNA) [12,13] and assessment of skeletal muscle mass, walking speed and hand grip strength.

Skeletal muscle mass (SMM) was measured by bioelectrical impedance analysis (Model InBody S20; InBody, Seoul, Korea). Skeletal muscle mass index (SMI) was calculated as SMM (kg) divided by height (meters)^2^. Turkish population SMI cut-off values (9.2 kg/m^2^ and 7.4 kg/m^2^ in males and females, respectively) previously determined by Bahat et al., were used to define low skeletal muscle mass index [14]. Muscle strength was measured by using hand grip dynamometer (T.K.K.5401; Takei Scientific Instruments, Tokyo, Japan) while the patient was standing arms parallel to the floor. Three consecutive measurements were made holding the instrument in the dominant hand. The highest of the three measurements was taken for analysis. Walking time (s) was assessed with 4.6-meter walking test by using a manual stopwatch. Walking speed (m/s) was calculated by dividing 4.6 m to the walking time (s) of 4.6 m.

### 2.2. Frailty assessment

FFP, modified FFP and frailty status clinically defined by the expert geriatrician after performing comprehensive geriatric assessment were used to determine the frailty status of the patients.

#### 2.2.1. FFP

FFP was translated to Turkish by independent translators by using forward-backward translation method. First FFP was translated to Turkish by two native Turkish speakers who are fluent and experienced in medical science translation. All the authors checked the Turkish version of the manuscript. Then, the Turkish version was translated back to English by a native English speaker experienced in medical sciences and blinded to the original questionnaire. Two geriatricians rechecked the compliance between back translated and original form of the FFP and approved the latest Turkish version of the FFP. Turkish version of FFP was presented in Table 1 in supplementary file.

Fried frailty phenotype consists of five criteria: weight loss, exhaustion, physical inactivity, low hand grip strength, and slow walking speed. Patients who have three or more of these criteria are defined as frail, who have one or two criteria, are defined as prefrail and none of the criteria are defined as robust. Weight loss was identified as unintentional weight loss of 4.5 kg or 5% of body weight in the prior year. Exhaustion was determined by asking the questions from the Center for Epidemiologic Studies – Depression (CES–D) scale [15]: ‘How often in the last week you felt that everything you did was an effort?’ and ‘How often in the last week you felt that you could not get going?’ 0 = rarely or none of the time (1 day), 1= some or a little of the time (1–2days), 2 = a moderate amount of the time (3–4 days), or 3 = most of the time. Participants answering 2 or 3 either of these questions are identified as satisfying exhaustion criteria. Sedentary behavior was detected by Minnesota Leisure Time Physical Activity Questionnaire [16]. Energy expenditure less than 383 kcal/week for men and 270 kcal/week for women were defined as sedentary lifestyle or low-calorie expenditure [4]. Hand grip strength was determined by using hand grip dynamometer while the patients standing and their arms parallel to the floor and three consecutive measurements were taken in the dominant hand. The highest of the three measurements was recorded for analysis. Originally defined thresholds in Cardiovascular Health Study adjusted for gender and body mass index was used as cut-off thresholds. Patients have lower hand grip cut-offs than the determined thresholds were defined as low hand grip strength. Patients who have higher walking time than the defined walking time cut offs for 4.6 m adjusted for sex and height in FFP, were accepted as slow walking speed.

#### 2.2.2 Modified FFP

A modified FFP was created by rescoring FFP by using the hand grip strength cut-off values that best predict low SMI for men and women in Turkish population.

#### 2.2.3. Frailty clinically defined by expert physicians

Two clinicians experienced in geriatric medicine over three years, determined the frailty status of the patients as robust, prefrail, and frail by using the data consisting of the age, gender, anthropometric measurements, comorbid diseases, number of drugs, alcohol use, smoking status, comprehensive geriatric assessment test scores (ADL, IADL, MMSE, GDS, MNA) and walking speed, independently. Due to the good degree of compliance between the two clinicians’ decisions (kappa: 0.61; p < 0.001), the frailty assessment of more experienced clinician was adopted as the gold standard for this study.

### 2.3. Construct validity and reliability

For construct validity of FFP, it was compared with the gold standard, i.e. definition of frailty status by expert geriatrician, after comprehensive geriatric assessment. Inter and intra-clinician concordance were evaluated for reliability assessment. For interclinician concordance, two clinicians experienced in geriatric medicine, evaluated the frailty status of the 35 patients consecutively, in different rooms by using the Fried frailty phenotype. For intra-clinician concordance, FFP was reapplied to 35 patients with an interval of 1 week.

### 2.4. Statistical analysis

SPSS version 16 was used to perform statistical analyses. Descriptive statistics were presented as mean (SD) for normally distributed continuous variables or median (min-max) for nonnormally distributed ones and percentages (%) in case of categorical variables. The capacity of hand grip strength values in predicting low skeletal muscle mass index were analyzed using ROC curve analysis. When a significant cut-off value was determined, the sensitivity, specificity, positive and negative predictive values were presented. Interrater and intra-rater agreement and agreement between clinician’s assessment and FFP or modified FFP was investigated using Cohen’s Kappa test. P value less than or equal to 0.05 was accepted as statistically significant.

## 3. Results

A total of 450 patients, aged 59 years and over, were included. Mean (SD) age was 75.45 (6.70). 61.3% of the patients were female. The three most frequent comorbidities were hypertension (71.3%), diabetes mellitus (33.1%), and coronary artery disease (25.6%).

**Figure 1:**
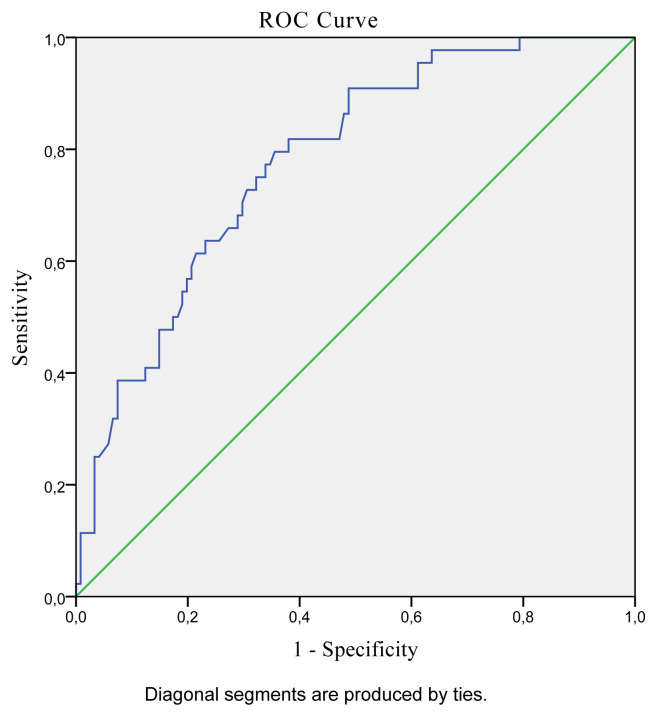
ROC curve analysis of hand grip strength predicting low SMI for men.

**Figure 2:**
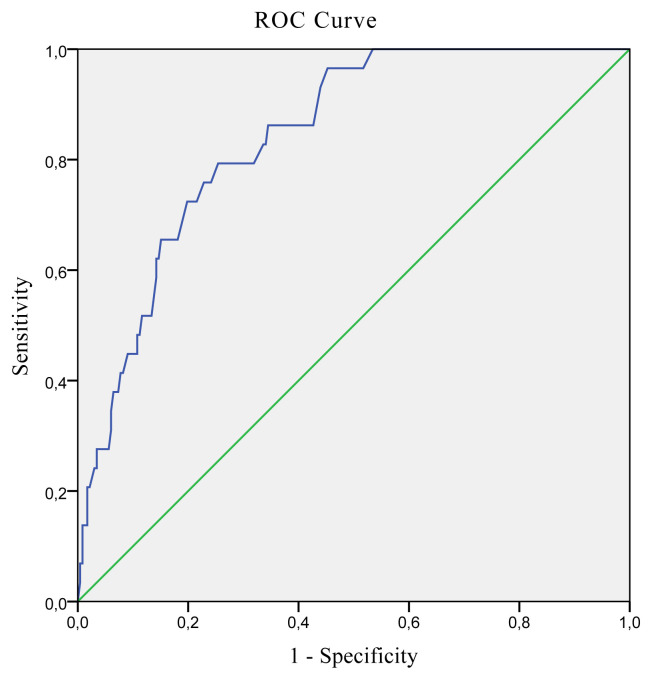
ROC curve analysis of hand grip strength predicting low SMI for women.

The best hand grip strength cut-off values predicting low SMI in older Turkish population were determined as ≤13.6 kg for women (AUC: 0.841; 95% CI: 0.791–0.883; Sensitivity: 79.31; Specificity: 74.57; p < 0.001) and ≤27.7 kg for men (AUC: 0.779; 95% CI: 0.708–0.840; Sensitivity: 79.55; Specificity: 64.46; p < 0.001). ROC curves presenting the best hand grip strength cut-off values predicting low SMI for men and women are presented in Figure 1 and 2, respectively.

According to FFP, 25.6% of the patients were robust, 49.0% were prefrail and 25.4% were frail. Frailty status of the patients determined by FFP, modified FFP, and clinician’s frailty assessment are presented in Table 1. Results of comprehensive geriatric assessment parameters of the patients categorized by clinician’s frailty assessment are presented in Table 2.

When patients were categorized as robust, prefrail or frail; a good concordance was found between the clinician’s frailty assessment and FFP (kappa 0.66; p < 0.001). Modified FFP had good agreement with the FFP (kappa: 0.70, p < 0.001). Interclinicians and intra-clinician compliance were good (kappa: 0.67, p < 0.001 and kappa 0.74, p < 0.001, respectively).

When patients were categorized as frail or not frail; good correlation between clinician’s frailty assessment and modified FFP was observed (kappa: 0.73; p < 0.001). An excellent agreement was found between FFP and modified FFP (kappa: 0.84 and p < 0.001) and between clinicians’ assessments and FFP (kappa: 0.84 and p < 0.001).

Concordance between the clinicians’ assessments and FFP and modified FFP are presented in Table 3. Intra-clinician and interclinicians’ consistencies are presented in Table 3.

## 4. Discussion

In this study, the validity and reliability of FFP and modified FFP (modified by using the hand grip cut-offs for Turkish population) in the Turkish population were investigated. The best hand grip strength cut-off values predicting low SMI in older Turkish population were determined as ≤13.6 kg for women and ≤27.7 kg for men.

A good concordance was found between the clinician’s frailty assessment and FFP when patients were categorized as robust, prefrail, and frail. Modified FFP had good agreement with the original FFP. In addition, good concordance between clinician’s frailty assessment and modified FFP was observed. Interrater and intra-rater agreements were good. These results support that FFP, as well as modified FFP are valid and reliable tools for detecting frail older adults in Turkish population.

Frailty is a common multidimensional condition consisting of physical, psychological, and social components. Frailty prevalence in Turkey is reported to be 15.4%–27.8% in community dwelling older adults and 39.2% in Physical Medicine and Rehabilitation outpatient clinics (REF) [17,18]. Frailty is an important risk factor for disability and mortality in older adults and it can be reversed by proper clinical management. Therefore, frailty assessment is an indispensable component of determining the medical care plans of older patients.

Comprehensive geriatric assessment is accepted as gold standard method in identifying frailty. In busy clinical practice validated frailty indexes is preferred for detecting frailty status. FFP is one of the most common used frailty indexes that is based on physical frailty assessment. FFP has four objective criteria, in which one of them is hand grip assessment. Hand grip cut-offs by sex and BMI can vary across different populations. In our study, we used the Turkish population SMI cut-offs (9.2 kg/m2 and 7.4 kg/m2 for males and females, respectively) to detect the best hand grip strength cut-off values predicting low skeletal muscle mass index. We calculated hand grip cut-offs as ≤13.6 kg for women and ≤27.7 kg for men in older Turkish population in this study. Bahat et al. have defined the cut-off thresholds of hand grip strength (cut-off values that predicted gait speed <0.8 m/s) as 32 kg and 22 kg for males and females, respectively, in Turkish population [14]. These hand grip strength cut-offs are higher compared to the hand grip cut-offs in our study and cut-offs in FFP. In our study hand grip strength cut-offs were based on the best predicting values for low SMI, instead of walking speed. Neurological problems and joint diseases like advanced osteoarthritis can affect walking speed, for this reason, we preferred to use hand grip cut-offs that predict low SMI instead of walking speed. In another study in Turkish population, Bulut et al. have defined hand grip strength thresholds as 14 kg in women and 28 kg in men according to the two SD below the mean of healthy young participants [19]. Our hand grip thresholds are comparable with these results [19]. These hand grip cut-offs might be more suitable for predicting low SMI in Turkish older population.

This study has some strengths. Our sample size is large, and they all underwent a comprehensive geriatric assessment that also included frailty and sarcopenia assessments. Moreover, this is the first study that investigated the cross-cultural validation of one of the most used frailty scales, FFP. In addition, this is the first study in which hand grip thresholds that are best predictive for low SMI according to sex for Turkish older patients are determined.

The limitation of this study is having a cross-sectional design. Therefore, for validation, only the consistency between clinician’s decision and FFP and modified FFP were assessed. The long-term predictive ability of these frailty assessment methods for disability or mortality could not be evaluated. For this sense, prospective studies to elucidate the predictive value of FFP and modified FFI on disability or mortality are needed.

## 5. Conclusion

This study results suggest that FFP is a valid and reliable index for Turkish population. Using modified cut-offs does not seem to improve agreement with the clinically defined frailty status, however, further prospective studies are needed to explore its value in predicting morbidity and mortality.

## Supplementary Information



## Figures and Tables

**Table 1:** Frailty status according to the FFP, Modified FFP, and clinician’s assessment.

	Robust (%)	Prefrail (%)	Frail (%)
FFP	25.6	49.0	25.4
Modified FFP	39.0	40.6	20.4
Clinician’s assessment	30.6	40.4	29.0

**Table 2:** Results of comprehensive geriatric assessment parameters of the patients categorized by clinician’s frailty assessment.

	Robust	Prefrail	Frail
Age, year, median (min–max)	72 (65–87)	76 (59–91)	79 (65–97)
Gender, female, n (%)	61 (48.8)	109 (66.1)	81 (68.6)
Alcohol use, n (%)	3 (2.4)	4 (2.4)	1 (0.8)
Smoking, n (%)	10 (8)	12 (7.3)	5 (4.2)
Diabetes Mellitus, n (%)	35 (28)	72 (43.6)	30 (25.4)
Hypertension	77 (61.6)	125 (75.8)	90 (76.3)
Chronic obstructive pulmonary disease, n (%)	5 (4)	6 (3.6)	5 (4.2)
Congestive heart failure, n (%)	7 (5.6)	13 (7.9)	17 (14.4)
Number of drugs, median (min–max)	4 (0–15)	6 (0–15)	6 (0–15)
BMI (kg/m2), median (min–max)	28.3(19.0–45.0)	28.5 (17.5–48.8)	27.2 (16.4–46.7)
ADL, median (min–max)	6 (5–6)	6(1–6)	5(0–6)
IADL, median (min–max)	8 (6–8)	8(1–8)	4(0–8)
MMSE, median (min–max)	28 (19–30)	26(8–30)	21(0–30)
GDS, median (min–max)	1 (0–6)	4(0–14)	6(0–15)
MNA, median (min–max)	14 (11–14)	12(5–14)	8(3–12)
SMI, mean (SD)	9.9 (1.31)	9.3 (1.21)	8.6 (1.36)
Hand grip (kg), median (min–max)	24 (10.9–48.3)	20.0(5.8–41.7)	14.6(0–33.6)
Walking speed, (m/s), median (min–max)	1.38 (0.48–2.30)	1.15 (0.27–2.00)	0.56 (0.12–1.47)
Calf circumference (cm), median (min–max)	37 (29.5–49.0)	36(27–49)	34(27–47)

**Table 3:** Concordance between the frailty assessments.

	Robust/prefrail/frail		Frail /not-Frail		Robust/not Robust	
	Kappa	p	Kappa	p	Kappa	p
Clinician’s assessment & FFP	0.66	<0.001	0.84	<0.001	0.60	<0.001
Clinician’s assessment &Modified FFP	0.51	<0.001	0.73	<0.001	0.51	<0.001
FFP & Modified FFP	0.70	<0.001	0.84	<0.001	0.68	<0.001
Inter-rater	0.67	<0.001	0.46	0.006	0.87	<0.001
Intra-rater	0.74	<0.001	0.78	<0.001	0.72	<0.001
