# Supplementary material for: Validity and reliability of Fried frailty phenotype in Turkish population
Source: Turk J Med Sci. 2021 Oct 23;52(2):323–8. doi: 10.3906/sag-2105-165 (PMC10381207; doi:10.3906/sag-2105-165)
Supplement: Supplementary file 1 [file SAG-2105-165_2_Table1_supplementary_file.docx]

Supplementary Table1. Turkish Version of Fried Frailty Phenotype

| Fried Kırılganlık Fenotipi | |
| --- | --- |
| **1.Kilo kaybı** | "Geçen yıl, istemsiz (diyet veya egzersiz sebebiyle olmayan) 4,5 kilogramdan veya ağırlığınızın %5’inden daha fazla kilo kaybınız oldu mu?" Evet ise, kilo kaybı kriteri için kırılgan olarak tanımlanır. |
| **2**.**Tükenmişlik:** | CES-D Depresyon Ölçeğindeki iki ifade okunur.  (a) Yaptığım her şeyde çaba harcadığımı hissediyorum.  (b) Devam edemiyorum.  ʺGeçen hafta ne sıklıkta böyle hissettiniz? ʺ sorusuna  **0** = nadiren veya hiçbir zaman (<1 gün),  **1** = Biraz veya az bir kısmında (1-2 gün),  **2** = Bazen (3-4 gün) **3** = çoğu zaman.  İki sorudan birine "**2**" veya "**3**" cevabı veren kişiler, tükenme kriteri açısından kırılgan olarak kategorize edilir. |
| **3**. **Fiziksel Aktivite** | Minnesota Boş Zaman Etkinliği anketinin kısa versiyonuna göre yürüyüş, ev işleri (orta derecede yorucu), çim biçme, bahçe işleri yapma, yürüyüş, koşu, bisiklet sürme, dans etme, aerobik, bowling, golf, tek başına tenis, karşılıklı tenis, raket topu, jimnastik, yüzme aktiviteleri hakkında sorulur. Aktivitelere harcanan haftalık kaloriler standart algoritma kullanılarak hesaplanır. Bu değişken cinsiyete göre sınıflandırılır.  **Erkekler:** Haftada 383 Kcal 'den az fiziksel aktivite gösterenler bu kriter için kırılgan olarak değerlendirilir.  **Kadınlar:** Haftada 270 Kcal 'den az fiziksel aktivite gösterenler bu kriter için kırılgan olarak değerlendirilir. |
| **4.** **Yürüme süresi:** | **(Cinsiyet ve boya göre sınıflandırılmış)**  ***Cinsiyet ve boya göre 4,6 metre yürüme süresi için sınır değerler***  ***Erkekler***  Boy ≤ 173 cm ≥7 saniye  Boy> 173 cm ≥6 saniye  ***Kadınlar***  Boy ≤ 159 cm ≥7 saniye  Boy> 159 cm ≥6 saniye |
| **5.El kavrama gücü:** | **(Cinsiyet ve vücut kütle indeksi (VKİ) temel alınarak sınıflandırılmış)**  *Kırılganlık için el kavrama gücü (Kg) sınır değerleri:*  ***Erkekler***  VKİ ≤ 24 ≤29  VKİ 24,1-26 ≤30  VKİ 26,1-28 ≤30  VKİ > 28 ≤32  ***Kadınlar***  VKİ ≤ 23 ≤17  VKİ 23,1-26 ≤17,3  VKİ 26,1-29 ≤18  VKİ > 29 ≤21 |
